# Aquaporins in Immune Cells and Inflammation: New Targets for Drug Development

**DOI:** 10.3390/ijms22041845

**Published:** 2021-02-12

**Authors:** Inês V. da Silva, Graça Soveral

**Affiliations:** 1 Research Institute for Medicines (iMed.ULisboa), Faculty of Pharmacy, Universidade de Lisboa, 1649-003 Lisboa, Portugal; imsilva1@campus.ul.pt; 2 Department of Pharmaceutical Sciences and Medicines, Faculty of Pharmacy, Universidade de Lisboa, 1649-003 Lisboa, Portugal

**Keywords:** aquaporins, immune cells, immunity, inflammation, inflammatory disease

## Abstract

The mammalian immune system senses foreign antigens by mechanisms that involve the interplay of various kinds of immune cells, culminating in inflammation resolution and tissue clearance. The ability of the immune cells to communicate (via chemokines) and to shift shape for migration, phagocytosis or antigen uptake is mainly supported by critical proteins such as aquaporins (AQPs) that regulate water fluid homeostasis and volume changes. AQPs are protein channels that facilitate water and small uncharged molecules’ (such as glycerol or hydrogen peroxide) diffusion through membranes. A number of AQP isoforms were found upregulated in inflammatory conditions and are considered essential for the migration and survival of immune cells. The present review updates information on AQPs’ involvement in immunity and inflammatory processes, highlighting their role as crucial players and promising targets for drug discovery.

## 1. Introduction

Mammalians use both innate and adaptive mechanisms to detect and eliminate all kinds of pathogens. Such a process is resolved by inducing inflammation as a mechanism of tissue clearance. This review presents the state of the art concerning aquaporins’ (AQPs) role in immune- and inflammatory-related biological processes such as cell–cell communication, migration and phagocytosis that are crucial for achieving cellular immune response. Targeting AQPs in immune cells will boost the design of novel drugs and paves the way for the development of new therapies.

A literature search was undertaken using various online sources including PubMed and the Web of Science platform/database, and results were generated by the combination of “Aquaporin” and one of the following keywords: “inflammation”, “inflammatory”, “immune” and “immunity”. Using the available data, we focused on the role of AQPs in inflammation, shedding light on their potential targeting to overcome inflammation and related pathologies and encouraging the search in drug discovery.

## 2. Aquaporins

The cellular and molecular events associated with inflammation are complex, and therefore, every protein that plays a part in such mechanisms should be considered. In recent years, AQPs have been raised as relevant players in both immune cell physiology and inflammatory response, opening new perspectives for innovative therapeutics.

AQPs are channel-forming proteins with representation in all kinds of organisms [1]. AQPs facilitate the transport of water and small non-charged molecules through the plasma membrane, driven by osmotic and solute gradients [2,3,4]. Despite being part of the same family, the 13 (AQP0–12) isoforms expressed in humans present specific permeability features and tissue/subcellular localization, suggesting a link between the site of expression and function [3,5]. The orthodox aquaporins (AQP0, AQP1, AQP2, AQP4, AQP5, AQP6 and AQP8) are highly selective to water, having a crucial role in transepithelial water transport to maintain fluid homeostasis, while aquaglyceroporins (AQP3, AQP7, AQP9 and AQP10) also transport small non-charged solutes such as glycerol and urea, thus having high impact in energy balance with implications in metabolism [5,6]. S-aquaporins (AQP11 and AQP12) are two subcellular isoforms—AQP11 facilitates water and glycerol transport across plasma and organelle membranes, guaranteeing intracellular homeostasis in several organs, while AQP12 permeability still needs investigation [7,8,9,10,11]. Additionally, peroxiporins (AQP3, AQP5, AQP8, AQP9 and AQP11), due to their ability to facilitate hydrogen peroxide permeation through membranes regulating hydrogen peroxide fluxes [12,13,14,15,16], are tightly involved in redox balance and are emerging as important players in immunity, with possible implications in oxidative stress and inflammation.

In recent results, mainly from knockout (KO) mice, AQPs have been associated with a variety of important physiological roles including transepithelial fluid transport, brain water homeostasis, osmoregulation, cell migration and proliferation [17,18] and have been suggested as potential targets for drug development [5,6].

## 3. Aquaporins in Immune Cells’ Physiology and Inflammation

The cells that constitute the immune system—lymphocytes (thymus cells, bone marrow cells and natural killer cells), neutrophils and monocytes—have the ability to act fast when they sense danger signals since they can undergo rapid morphological modifications. This depends on their capacity to alter the cytoskeleton structure by modulating water and specific small solutes’ permeability across the plasma membrane [19].

Based on the reported interaction between AQPs, the cytoskeleton and signaling cascades, AQPs’ involvement in the development of inflammatory mechanisms has recently been suggested, supported by the detection of several isoforms in cells of both innate and adaptive immunity and the demonstration of their dysregulation in various human diseases [20,21]. So far, AQPs have been described in specific processes related to immune cell function such as priming and inflammasome activation, transendothelial migration and phagocytosis [22,23,24,25,26].

### 3.1. Aquaporins in Immune Cell Priming

AQPs are expressed in human immune cells in both the innate and the adaptive immune system, and signals for cell activation/priming were shown to upregulate AQP isoforms during this process. In human blood leukocytes, AQP1 and AQP9 were detected and were upregulated after intravenous or in vitro lipopolysaccharide (LPS) stimulation [27,28]. AQP9 is also augmented in activated polymorphonuclear leukocytes in patients with systemic inflammatory response syndrome [29] and infective endocarditis [30]. Activated B and T lymphocytes were reported to express AQP1, AQP3 and AQP5 and immature dendritic cells (DCs) express AQP3 and AQP5, and their expression was related to activation and proliferation of these immune cells [21].

**Figure 1:**
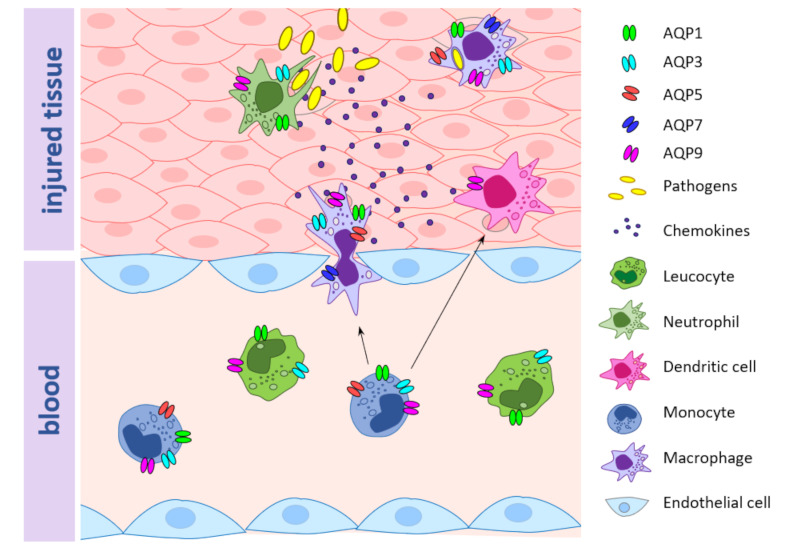
Aquaporins (AQPs) expression in immune cells involved in the inflammatory process. Illustration represents an injured tissue, adjacent blood vessel and immune cells involved in inflammation. The localization of each AQP isoform in the different immune cells is also represented.

In DCs, AQP9 was shown to be the most expressed isoform and was significantly upregulated by LPS stimulation. In AQP9-KO mice with induced colitis, AQP9 blockage did not completely protect from colitis-related inflammation but reduced DC inflammatory response [31]. Human primary blood-derived macrophages and neutrophils are characterized by high levels of AQP9, whose upregulation at both transcript and protein levels was also detected after stimulation with LPS [32]. Similar to primary cells, AQP3 is upregulated by LPS stimulation in monocytic THP-1 cells, which are commonly used as a model to study the inflammatory process. In THP-1 cells, AQP3 inhibition or silencing partially blocks LPS priming and decreases production of interleukin (IL)-6, pro-IL-1β, and tumor necrosis factor alpha (TNF-α), suggesting a link between AQP3 function and Toll-like receptor 4 (TLR4) engagement during macrophage priming [32]. Another study on THP-1 cells reported an increase in AQP1 expression after LPS administration, while AQP5 mRNA expression was reduced [33]. In addition, patients with systemic inflammatory response syndrome (SIRS) show increased AQP9 expression in neutrophils compared to healthy controls [29]. Figure 1 and Table 1 summarize the reported regulation of immune-related AQPs during inflammation.

### 3.2. Aquaporins in Inflammasome Activation

The inflammasome is an important player in the immune response—it can be found in macrophages and neutrophil granulocytes and recognize a variety of pathogen antigens. Inflammasome nucleotide-binding oligomerization family pyrin domain containing 3 (NLRP3) is upregulated in sepsis [34] and its activation modulates the release of pro-inflammatory cytokines such as IL-1β and IL-18 [35]. IL-1β release depends on extracellular pH and is caused by AQP-mediated water influx [36]. Therefore, AQP-mediated water movement in macrophages appears to be the common element unifying the variety of NLRP3 inflammasome activators [23]. In an acute lung injury mouse model, AQP1 deficiency was associated with a reduction in IL-1β release and neutrophilic inflammation, suggesting that AQP-mediated water transport in macrophages constitutes a danger signal required for NLRP3 activation [23].

**Figure 2:**
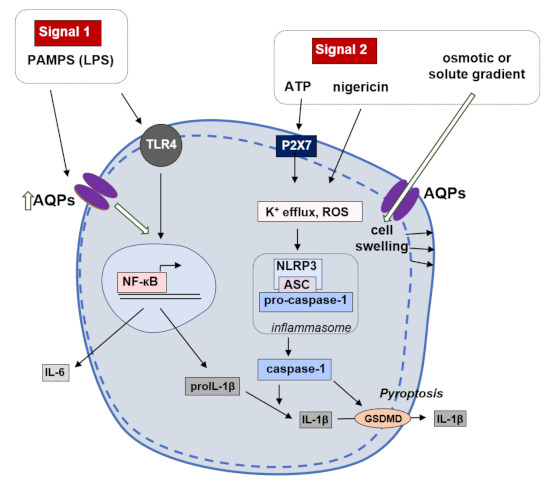
NLRP3-inflammasome priming and activation in macrophages. Two signals regulate the activation of NLRP3 inflammasome. During signal 1 (cell priming), Toll-like receptor 4 (TLR4) activation triggers nuclear factor (NF)-кB, enhancing the expression and synthesis of pro-inflammatory cytokines (interleukin (IL)-6, pro-IL-1β and pro-IL-18). Signal 2 (inflammasome activation) promotes the assembly of the inflammasome components. Pro-caspase-1 is recruited and activated, thus being able to process pro-IL-1β and pro-IL-18 to their mature and active forms, IL-1β and IL-18, respectively. Caspase-1 also promotes plasma membrane pore formation and consequent release of cytokines and cell death by pyroptosis.

More recently, fast cell reswelling has been pointed out as an event preceding macrophage activation and consequent IL-1β secretion [37,38,39]. In line with this, our previous study demonstrated that aquaporin-dependent cell reswelling increased IL-1β release through caspase-1 activation. Moreover, blockage of AQP3, which transports glycerol and hydrogen peroxide and is the most expressed isoform in monocytic THP-1 cells, reduced IL-1β release and pyroptosis by preventing inflammasome activation induced by reswelling, nigericin and ATP [32]. Thus, AQP3 overexpression may account for the fast cell volume changes occurring in inflammation. In addition, AQP3 peroxiporin activity contributing to rising intracellular reactive oxygen species (ROS) with subsequent inflammasome activation should also be considered. The proposed mechanism of inflammasome priming and activation in macrophages where AQPs may play a pivotal role is represented in Figure 2.

### 3.3. Aquaporins in Cell Shape-Dependent Mechanisms

AQPs mediate cell shape changes in several physiological processes required for cellular immunity such as migration, phagocytosis and antigen uptake by interacting with the cytoskeleton and signaling cascades [40].

DCs express AQP5 and AQP7, and their ablation was associated with decreased antigen uptake and reduced endocytosis ability [41,42]. Studies in DCs of AQP7 KO mice showed that AQP7 is involved in chemokine-dependent migration and antigen uptake and processing [42].

AQP3, AQP5 and AQP9 were suggested as the most relevant AQP isoforms in the immune system as they regulate the migration of different immune cells [43]. AQP3 was shown to have an essential role in T cell and macrophage function and migration in a chemokine gradient [44]. AQP3-mediated transport of H_2_O_2_ in CD8+ T cells was also suggested as an important regulator in endocytosis and the endosome-to-cytosol transfer process during antigen uptake [45]. AQP5 and AQP9 regulate neutrophil cell migration and impact sepsis survival [43].

In leukocytes, AQP9 was found localized on the cell edges, possibly to facilitate motility, lamellipodium extension and stabilization and cell volume changes, enabling these cells to move toward chemoattractants [46]. *Pseudomonas aeruginosa*-induced upregulation of AQP9 in human macrophages is accompanied by changes in macrophage size and morphology, affecting cell motility, migration and phagocytosis [25]. In virus-activated memory CD8+ T cells, but not naive cells, IL-7 induces AQP9 expression, which is required for long-term cell longevity and homeostasis [47]. In addition, in a murine model of skin allergic contact dermatitis using AQP9 KO mice, AQP9-deficient neutrophils’ recruitment is attenuated and migration ability is decreased. Furthermore, neutrophil deficiency in AQP9 KO mice induces decreased IL-17A production by draining lymph node cells, resulting in low T cell activation [48].

## 4. Involvement of Aquaporins in Inflammatory Diseases

**Figure 3:**
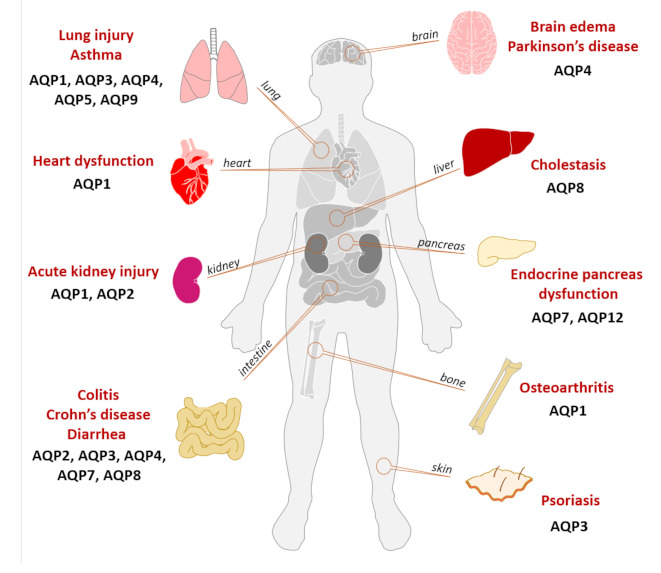
Involvement of AQPs in inflammatory diseases. Illustration of the various AQP isoforms involved in inflammation-associated diseases and respective affected organs.

Various animal models have been used to clarify the pathophysiology of inflammation-related diseases and understand the interplay between AQPs and the mechanisms underlying the inflammatory process. Figure 3 summarizes the involvement of various AQPs in inflammatory diseases, with special focus on the different organs affected.

### 4.1. Acute Lung Injury

Acute lung injury (ALI) is characterized by neutrophilic alveolitis, injury of the alveolar epithelium and endothelium, hyaline membrane formation and microvascular thrombi [49]. Different animal models of experimental ALI have been used to investigate mechanisms of lung injury and AQPs’ involvement in the process, and they can be achieved by submitting the animals to LPS, ventilation, hyperoxia or hydrochloric acid (HCl). Using rat ALI models, AQP1 was shown to be upregulated by LPS compared to control rats, and the same study reported that treatment with *Salvia miltiorrhiza* regulates the expression of AQP1, improving body fluid homeostasis and alleviating lung edema [50]. When inducing lung injury and edema by mechanical ventilation with high tidal volume, the expression of pulmonary AQP1 decreases [51]. Another study shows that AQP5 is strongly expressed in alveolar epithelial cells and is notably impaired after 3–14 days of hyperoxia treatment, suggesting that AQP5 is important for water movement in alveolar epithelial cells and its abnormal expression may lead to pulmonary edema [52].

The induction of lung inflammation in a murine model by LPS, HCl and ventilation yielded increased lung vascular permeability and inflammatory cell infiltration in the broncho-alveolar lavage fluid, and ventilation also induced altered lung mechanics. These data showed that involvement of AQPs in the acute inflammatory process is dependent on the localization and the type of lung injury. Among the AQP isoforms evaluated (AQP1, AQP4, AQP5 and AQP9), AQP4 lung expression decreased in the HCl- and ventilation-induced models that primarily targeted the alveolar epithelium, while AQP5 expression was impaired in the LPS-induced model targeting the capillary endothelium and alveolar epithelium [53]. Another study using an ALI mouse model focusing on the expression of AQP1, AQP3, AQP4 and AQP5 suggested AQP1 and AQP5 to play important roles in the abnormal fluid transport in ALI and their association with the development of pulmonary edema. AQP3 and AQP4 were not correlated with pulmonary edema during ALI [54].

### 4.2. Osteoarthitis

Osteoarthritis is a degenerative disease with an irreversible course, caused mainly by chondrocyte apoptosis and cartilage matrix degradation, which are pivotal players in regulating the function of articular cartilage by synthesizing the structural components of the extracellular matrix and matrix-degrading proteases. AQPs have been described in cartilage cells involved in fluid transport and in the regulation of cartilage physiology [55]. In Sprague Dawley rats whose osteoarthritis was surgically induced, resulting in severe cartilage damage, AQP1 expression was positively correlated with caspase-3 expression and activity, suggesting that AQP1 triggers caspase-3 activation, contributing to chondrocyte apoptosis and, consequently, to the development of osteoarthritis [56,57].

### 4.3. Neuroinflammation

AQP4 is the most representative AQP isoform in the brain and has been extensively studied in this tissue since it is involved in the pathophysiology of a variety of encephalopathies [58]. In a model where endotoxemia was induced in C57Bl/6 mice by intraperitoneal injection of LPS, resulting in severe central nervous system injury, AQP4 protein increased along with augmented cytokine release. Interestingly, dexamethasone attenuates AQP4 expression and IL-6 release, restoring the LPS inflammatory effect [59]. Furthermore, microRNA-130a was suggested as a therapeutic target/molecule since it inhibits AQP4 transcription in astrocytic end-feet, which, in turn, reduces astroedema and neuroinflammation [60]. AQP4 is also an astrocytic proteomic marker, since it was found upregulated in sepsis-induced delirium [61] and Parkinson’s disease [62]. AQP4 KO mice astrocyte-microglial co-cultures showed increased basal and inducible canonical NF-κB activity, enhanced gliosis (astrocytosis and microgliosis) and increased IL-1β and TNF-α release, suggesting that AQP4 deficiency promotes microglial cells’ activation in the co-culture system and mediates the communication between astrocytes and microglia [62]. Furthermore, studies in a rat model of cerebral edema showed that the activation of TLR4 and corticotropin-releasing hormone (CRH)/CRH receptor 1 (CRHR1) signaling upregulated AQP4 and water permeability in the brain during short hypoxia. In the same model, LPS treatment by itself increased AQP4 and pro-inflammatory cytokines, but brain edema was only accomplished by conjugating LPS treatment with hypoxia. Humans submitted to hypobaric hypoxia also showed enhanced TNF-α, IL-1β, IL-6 and CRH plasma levels. These data suggest that systemic inflammation facilitates the onset of hypoxic cerebral edema, where AQP4 plays an important role [63].

### 4.4. Asthma

Asthma is characterized by chronic inflammation of the airways. Oxidative stress plays a decisive role in the pathogenesis of asthma since reactive oxygen species such as H_2_O_2_ may initiate airway inflammation. AQP3, by facilitating H_2_O_2_ membrane permeation, potentiates ovalbumin-induced murine asthma by increasing chemokine production (CCL24 and CCL22) from alveolar macrophages and T cell trafficking. Additionally, AQP3-KO mice exhibit reduced airway inflammation compared to wild-type mice [64].

### 4.5. Bowel Diseases

AQPs play an important role in transcellular water movement, being crucial for water absorption in the colon. AQP3 and AQP8 were detected in the colon of Sprague Dawley rats, and their expression was decreased in 2,4,6-trinitrobenzene sulfonic acid (TNBS)-induced colitis, a model that mimics human Crohn’s disease histopathology. The downregulation of AQP3 and AQP8 was accompanied by an increase in intestinal inflammation and injury, suggesting that both AQP3 and AQP8 may be involved in the pathogenesis of inflammatory bowel disease [65]. AQP8 downregulation was also described in human Crohn’s disease and ulcerative colitis biopsies, suggesting that AQP8’s role as a H_2_O_2_ channel is involved in metabolism, and its downregulation may represent a defense mechanism against severe oxidative stress.

Colitis has been extensively associated with alterations in electrolyte, water transport and fluid fluxes, which contribute to the increased susceptibility to mucosal injury. A murine model of colitis induced by dextran sulphate presented decreased AQP4 and AQP8 gene and protein levels that correlated with significant alteration in colonic fluid secretion. Accordingly, patients with active ulcerative colitis, Crohn’s colitis or infectious colitis showed similar reduced AQPs expression, indicating that colonic injury is associated with downregulation of AQPs expression [66]. Interestingly, glycerol membrane transport was demonstrated as being crucial for enterocyte physiology. The AQP3-KO rat model of colitis induced by dextran sulphate or acetic acid developed more severe colonic hemorrhage, marked epithelial cell loss and death than observed in wild-type rats, and these symptoms were significantly reversed by oral glycerol uptake, increasing survival and reducing the severity of colitis, and suggesting that AQP3 is implicated in enterocyte proliferation due to its glycerol facilitator function [67]. In addition, a genome-wide comparison of gene expression in genetically susceptible animals that develop spontaneous colitis showed that despite most upregulated genes in experimental colitis are immune-related, AQP4 and the mitochondrial ribosomal protein L33 were also strongly upregulated. These results were confirmed in dextran sodium sulfate-treated mice with colitis [68].

Diarrhea consists in transepithelial hypersecretion of fluid in the gastrointestinal tract and poor water absorption in the colon. The regulation of transepithelial fluid transport is based on ion and water transport, the latter being facilitated by AQPs. Altered expression of AQPs in the colon is correlated with the severity of diarrhea in animals and humans [69]. A model of 5-fluorouracil (5-FU)-induced diarrhea in mice showed increased pro-inflammatory cytokines (TNF-α, IL-1β, IL-6, IL-17A and IL-22) correlating with decreased AQP4 and AQP8 mRNA throughout the entire colon compared to control mice [70].

AQP2 and AQP3 are important isoforms in the regulation of water fluxes in apical and lateral mucosal epithelial cells in colon and are upregulated in diarrhea. Diarrhetic mice treated with tannin extract, which presents anti-diarrheal properties, have decreased AQP2 and AQP3 expression along with decreased water loss in colon, ameliorating colon health. Additionally, studies in HT-29 cells showed that tannin-induced AQP2 and AQP3 downregulation is a consequence of suppressing the protein kinase A (PKA)/cyclic adenosine monophosphate response element binding protein (pCREB) signaling pathway [71].

### 4.6. Psoriasis

Psoriasis is a chronic inflammatory skin disease characterized by raised plaques, epidermal hyperplasia and infiltration of leukocytes into the skin [72]. AQP3 KO mice with IL-23-induced psoriasis showed that AQP3, a water/glycerol/hydrogen peroxide (H_2_O_2_) channel protein, is required for nuclear factor-κB (NF-κB) activation and signaling in keratinocytes and in the pathogenesis of psoriasis. The same authors also demonstrated that cellular import of H_2_O_2_ produced by membrane NADPH oxidase 2 (Nox2) in response to TNFα is facilitated by AQP3 and required for NF-κB activation by regulation of protein phosphatase 2A [73].

### 4.7. Metabolic Diseases

Water and glycerol movements are crucial for metabolic homeostasis, and their altered expression has been extensively related to metabolic disorders [6].

Metabolic diseases, such as obesity, have a negative impact in pancreas physiology. In a recent work, we studied obesity-induced inflammation in the pancreas using AQP7- and AQP12-silenced rat β-cells stimulated by TNFα and LPS. AQP7, the main aquaglyceroporin in endocrine pancreas and involved in insulin exocytosis, was impaired by TNFα along with a drastic reduction in insulin secretion. AQP7 was upregulated by LPS, whereas AQP12 was upregulated by both TNFα and LPS. Cells overexpressing AQP12 revealed lower levels of pro-inflammatory cytokines release, emphasizing AQP12 implication in inflammation [74].

Cholestasis is a condition where bile cannot flow from the liver to the duodenum. AQP8 is an AQP isoform that facilitates canalicular osmotic water movement during hepatocyte bile formation. Its involvement in the pathogenesis of cholestasis was evaluated using an LPS-induced cholestasis rat model. In such conditions, AQP8 protein was decreased. In addition, LPS seems to induce TNFα-mediated post-transcriptional downregulation of AQP8, affecting its function and suggesting a potential mechanism of the pathogenesis of cholestasis [75].

Chronic liver injury is associated with inflammation in several diseases such as viral infections, metabolic disorders, and nonalcoholic steatohepatitis. AQP3, an H_2_O_2_ channel, is expressed in macrophages and involved in their activation triggering the hepatic inflammatory process. Administration of anti-AQP3 monoclonal antibody to a mice model of CCl4-induced liver injury and fibrosis prevented liver injury by blocking AQP3-mediated H_2_O_2_ transport and consequent inhibition of macrophage activation [76].

Cardiac dysfunction commonly occurs in patients with septic shock. AQP1 is essential for water homeostasis and vascular health and studies in AQP1-KO mice showed that these animals suffer from cardiac hypertrophy [77]. Sepsis-associated impaired cardiac function was induced in an LPS-stimulated mice model. In this study LPS administration led to increased levels of AQP1 and pro-inflammatory genes as well as cardiac dysfunction in old mice suggesting a contribution for hearts dysfunction in aged subjects with septic endotoxinemia [78].

### 4.8. Kidney Injury

Acute renal failure is frequently associated to sepsis and is characterized by impaired urinary concentration, increased natriuresis and decreased glomerular filtration rate. LPS-induced endotoxemic animal models confirmed AQP2 downregulation after short exposure [79,80,81] and upregulation after a long exposure time [82] in kidney. Treatment with propofol of an LPS-stimulated rat model prevented downregulation of AQP2 while protecting renal function in sepsis [83]. Treating animals with alpha-Lipoic acid preserved AQP2 expression while decreasing levels of pro-inflammatory cytokines and protected against LPS-induced tubular dysfunction by suppression of apoptosis and inflammation [84]. AQP1-KO mice also suggested a role for AQP1 in kidney homeostasis since these animals are predisposed to enhanced endotoxemic renal injury with lower glomerular filtration and urine osmolality [84].

The involvement of AQP isoforms in the settings of inflammation-related pathologies is summarized in Table 2.

## 5. Aquaporins as Druggable Targets in Inflammation

The pathophysiological implication of AQPs in immunity and inflammation indicate that these membrane proteins are promising drug targets and that their regulation in immune cells represents a potential therapeutic approach for the modulation of the inflammatory process.

Although several AQP modulators have been reported and patented for use for diagnostic and therapeutic purposes [88,89], their lack of selectivity and toxic side effects have hampered application in clinical trials. As for AQP3 and AQP7 channel activity, the gold(III) bipyridyl compound Auphen [90] was shown to inhibit glycerol permeability in adipocyte [91] and monocytic cell lines, reverting cell priming, an essential mechanism for the development of the inflammatory process [32]. Recently, the commercially available compounds DFP00173 and Z433927330 were identified as new potent and selective AQP3 and AQP7 inhibitors [92] and were suggested to be useful in the investigation of AQPs in cytokine signaling. In addition, the compound HTS13286 was reported to block the passage of glycerol and urea through AQP9, thus impairing secretion of inflammatory cytokines [93]. Alternatively, AQPs’ expression can be modulated at the transcriptional level, as reported for cytokines (IL-7), leading to increased cell longevity and homeostasis [47].

Knowing that a variety of AQP isoforms are involved in immunity and inflammation, the design and discovery of new molecules with the ability to modulate the expression and function of specific AQPs is of utmost interest and would undoubtedly promise new therapeutic approaches. However, the protein structural conformation with channel pore access restrictions makes the molecule difficult to target and has challenged the development of AQP drug discovery [5]. The recent recognition of miRNA-targeted AQP modulation [94] also displayed an impact in inflammation-associated diseases, such as AQP1-targeted miR-126-5p ameliorating the dysfunction of alveolar fluid clearance [95] and AQP1-targeted miR-144-3p reducing lung epithelial cell apoptosis in a mouse model of acute lung injury [96]. Recently, the production of antibodies targeting the AQP channel [76,97] has tailored new perspectives for the development of specific AQP-based therapeutics. Compounds for treating autoimmune inflammatory diseases such as neuromyelitis optica (NMO) boosted the successful development of monoclonal antibodies as blockers of IgG-AQP4 for the prevention and treatment of NMO lesions [97]. In a recent study, an antibody anti-AQP3 raised to block AQP3-facilitated H_2_O_2_ and glycerol transport in liver-resident macrophages prevented liver injury in an experimental mouse model [76]. Therefore, AQP-based development of novel therapeutics to reduce inflammation in a variety of disorders remains a promising strategy.

## 6. Final Remarks

Inflammation is a complex mechanism that plays a central role in the maintenance of mammalian physiology. Compelling evidence strongly suggests that a few AQP isoforms are key regulators of inflammation, participating in cytokine and growth factors’ signaling pathways, possibly by mediating H_2_O_2_ permeability in addition to their role as water or glycerol channels. AQPs’ contribution to several essential cellular processes that are the basis of inflammation resolution, such as cell priming and inflammasome activation, migration, antigen uptake and phagocytosis, as well as their involvement in several models of inflammatory disease makes these membrane proteins promising targets for drug discovery. A deeper insight into the contribution of immune-related AQPs to cellular and molecular mechanisms underlying inflammation may foster innovative therapeutics to treat inflammation. Validation of aquaporin-targeted therapies relies on the development of potent and selective AQP modulators, such as small molecules or biologics, and, hopefully, translation of the experimental data into clinical practice.

## Figures and Tables

**Table 1:** Regulation of immune-related AQPs during inflammation.

Gene	Species	Immune Cells	Stimuli	Regulation	References
AQP1	Human	Leucocytes	LPS	Upregulation	[28]
	Human	Monocytic THP-1 cells	LPS	Upregulation	[33]
AQP3	Human	Leucocytes	Sepsis	Downregulation	[28]
	Human	Monocytic THP-1 cells	LPS	Upregulation	[32]
AQP5	Human	Monocytic THP-1 cells	LPS	Downregulation	[33]
AQP7	Mouse	Macrophages		?	[24]
AQP9	Human	Leucocytes	SIRS	Upregulation	[29]
	Mouse	Dendritic cells	LPS	Upregulation	[31]
	Human	Macrophages	*Pseudomonas aeruginosa*	Upregulation	[25]
	Human	Leucocytes	LPS	Upregulation	[27]
	Human	Monocytes	LPS	Upregulation	[32]
	Mouse	Macrophages		?	[24]

AQP, aquaporin; LPS, lipopolysaccharide; SIRS, Systemic Inflammatory Response Syndrome.

**Table 2:** AQPs’ involvement in inflammatory diseases.

Gene	Animal Model	Tissue	Stimuli	Effect on AQPs	Disease/Condition	Reference
AQP1	Rat	Lung	LPS	↑	Lung injury	[50]
	Rat	Lung	Ventilation	↓	Lung injury	[51]
	Mouse	Lung	LPS	↓	Lung injury	[54]
	Mouse	Heart	LPS	↓	Heart dysfunction	[78]
	Mouse	Kidney	LPS	↓	Acute kidney injury	[85]
	Rat	Bone	Ligament/meniscus resection	↑	Osteoarthritis	[56,57,86]
AQP2	Mouse	Intestine	MgSO_4_	↑	Diarrhea	[71]
	Rat	Kidney	*Escherichia coli* endotoxin	↓	Acute kidney injury	[81]
	Rat	Kidney	LPS	↓/↑/↑	Acute kidney injury	[80,83,84]
AQP3	Mouse	Lung	Ovalbumin	↑	Asthma	[64]
	Rat	Intestine	TNBS	↓	Colitis	[65]
	Mouse	Colon	DSS	↓	Colitis	[67]
	Mouse	Intestine	MgSO_4_	↑	Diarrhea	[71]
	Mouse	Skin	IL-23	↑	Psoriasis	[73]
AQP4	Mouse	Lung	HCl; Ventilation	↓	Lung injury	[53]
	Rat	Brain	hypoxia	↑	Brain edema	[63]
	Mouse	Brain	LPS	↑	Sepsis	[59]
	Mouse	Brain	MPTP/probenecid PD model	↓	Parkinson’s disease	[62]
	Mouse	Colon	DSS	↓	Colitis	[66]
	Mouse	Caecum	DSS	↑	Colitis	[68]
	Mouse	Intestine	5-FU	↓	Diarrhea	[70]
AQP5	Rat	Lung	Hyperoxia	↓	Lung injury	[52]
	Mouse	Lung	LPS	↓	Lung injury	[53]
	Mouse	Lung	LPS	↓	Lung injury	[54]
AQP7	Mouse	Colon	DSS	↓	Colitis	[66]
	Rat	Endocrine pancreas	LPS/ TNFα	↓/↑	Endocrine pancreas dysfunction	[74]
AQP8	Rat	Colon	TNBS	↓	Colitis	[65]
	Mouse	Colon	TNBS, DSS, CD4CD4RB transfer	↓	Colitis	[87]
	Mouse	Colon	DSS	↓	Colitis	[66]
	Mouse	Intestine	5-FU	↓	Diarrhea	[70]
	Rat	Liver	LPS	↓	Cholestasis	[75]
AQP9	Mouse	Lung	LPS; Ventilation	↑	Lung injury	[53]
AQP12	Rat	Endocrine pancreas	LPS; TNFα	↑	Endocrine pancreas dysfunction	[74]

AQP, aquaporin; LPS, lipopolysaccharide; TNBS, 2,4,6-trinitrobenzene sulfonic acid; DSS, dextran sulfate sodium; IL-23, interleukin-23; MPTP, 1-metil-4-fenil-1,2,3,6-tetraidropiridina; PD, Parkinson’s disease; 5-FU, fluorouracil; CD4, cluster of differentiation 4; CD4RB, receptor linked protein tyrosine phosphatase encoding B determinant; TNFα, tumor necrosis factor-alpha; ↓, dowregulation; ↑, upregulation.

## Abbreviations

ALI	Acute lung injury
AQPs	Aquaporins
DC	Dendritic cells
H_2_O_2_	Hydrogen peroxide
IL	Interleukin
LPS	Lipopolysaccharide
NF-κB	Nuclear factor-κB
NLRP3	Nucleotide-binding oligomerization family pyrin domain containing 3
SIRS	Systemic inflammatory response syndrome
TLR4	Toll-like receptor 4
TNFα	Tumor necrosis factor alpha
